# Expression of hepatic stellate cell activation-related genes in HBV-, HCV-, and nonalcoholic fatty liver disease-associated fibrosis

**DOI:** 10.1371/journal.pone.0233702

**Published:** 2020-05-22

**Authors:** Lu He, Hui Yuan, Junjie Liang, Jian Hong, Chen Qu

**Affiliations:** 1 Department of Abdominal Surgery, Integrated Hospital of Traditional Chinese Medicine, Southern Medical University, Guangzhou, Guangdong, China; 2 Department of Hepatobiliary Surgery, The First Affiliated Hospital of Jinan University, Guangzhou, Guangdong, China; 3 Department of Pathophysiology, School of Medicine, Jinan University, Guangzhou, Guangdong, China; Harvard Medical School, UNITED STATES

## Abstract

Liver fibrosis is a manifestation of chronic liver injury. It leads to hepatic dysfunction and is a critical element in the pathogenesis of cirrhosis and hepatocellular carcinoma. The activation of hepatic stellate cells (HSC) plays a central role in liver fibrogenesis of different etiologies. To elucidate the molecular mechanism of this phenomenon, it is important to analyze the changes in gene expression that accompany the HSC activation process. In this study, we isolated quiescent and activated HSCs from control mice and mice with CCl_4_-induced liver fibrosis, respectively, and performed RNA sequencing to compare the differences in gene expression patterns between the two types of HSCs. We also reanalyzed public gene expression data for fibrotic liver tissues isolated from patients with HBV infection, HCV infection, and nonalcoholic fatty liver disease to investigate the gene expression changes during liver fibrosis of these three etiologies. We detected 146 upregulated and 18 downregulated genes in activated HSCs, which were implicated in liver fibrosis as well. Among the overlapping genes, seven transcription factor-encoding genes, *ARID5B*, *GATA6*, *MITF*, *PBX1*, *PLAGL1*, *SOX4*, and *SOX9*, were upregulated, while one, *RXRA*, was downregulated. These genes were suggested to play a critical role in HSC activation, and subsequently, in the promotion of liver fibrosis. We undertook the RNA sequencing of quiescent and activated HSCs and analyzed the expression profiles of genes associated with HSC activation in liver fibrotic tissues from different liver diseases, and also aimed to elucidate the changes in gene expression patterns associated with HSC activation and liver fibrosis.

## Introduction

Liver fibrosis, which is characterized by the excessive deposition of the extracellular matrix (ECM) in the liver [1], generally occurs in association with chronic liver injury induced by multiple factors, such as chronic hepatitis B virus (HBV) or hepatitis C virus (HCV) infection, alcohol abuse, and nonalcoholic steatohepatitis (NASH) [2, 3]. Progressive liver fibrosis impairs hepatic function and results in the incidence of cirrhosis or hepatocellular carcinoma [4]. Unlike liver cirrhosis, liver fibrosis is a reversible process. The removal of the fibrotic response-causing agent may aid the regression of fibrosis [2]. However, no drug has been approved for the treatment of liver fibrosis yet [5]; this explains the urgent need for the development of anti-fibrotic drugs.

As the primary source of ECM in the liver, activated hepatic stellate cells (HSCs) have been widely considered as a potential therapeutic target in liver fibrosis [6, 7]. In a normal liver, HSCs are quiescent and located in the space of Disse as retinoid storage cells [8]. During liver fibrosis, chronic liver injury or stimuli activate the quiescent HSCs, and subsequently trigger ECM production and accumulation, which eventually leads to liver fibrosis [9]. However, the pathological changes caused by different liver diseases are non-identical. HSC activation is a process common in liver fibrosis induced by various hepatic injures [6, 10, 11]. As the key link to liver fibrosis, it is particularly important to determine the changes in gene expression associated with HSC activation. However, there are limited sources of public high-throughput gene expression data on activated and quiescent HSCs.

In this study, we isolated primary quiescent and activated HSCs from control mice and mice with CCl_4_-induced liver fibrosis, respectively, and performed transcriptome sequencing to analyze the gene expression changes associated with HSC activation. Additionally, we reanalyzed public transcriptome data from HBV-, HCV-, and nonalcoholic fatty liver disease (NAFLD)-associated fibrotic liver tissues and integrated these data with our transcriptome sequencing data to determine the key regulatory genes associated with HSC activation in liver fibrosis of different etiologies.

## Material and methods

### Ethics statement

The experiments were performed in accordance with the Animal Ethics Procedures and Guidelines of the People’s Republic of China. All efforts have been made to alleviate suffering. The mice were housed under a controlled temperature (20±2°C) with 12-h light/12-h dark cycles and with free access to food and water. The mice were sacrificed by cervical dislocation, and 2% pentobarbital sodium was used for anesthesia. The experimental protocol was approved by the Animal Care and Use Committee of Southern Medical University.

### Cell culture

LX-2 cells were cultured in high-glucose DMEM medium with 10% fetal bovine serum (FBS), 100 U/mL of penicillin, and 100 μg/mL of streptomycin. The cells were maintained in a 5% CO_2_/water-saturated incubator at 37°C. The LX-2 cells were obtained from the cell bank of Central South University.

For TGF-β stimulation experiments, after incubation in serum-free medium for 24 h, LX-2 cells were respectively cultured in serum-free medium or serum-free medium containing 5ng/ml recombinant human TGF-β1 (Sino Biological, Beijing, China). Cells were lysed after 24h for RNA isolation.

### CCl_4_-induced mouse liver fibrosis model

Six weeks old C57BL/6 male mice (Guangdong Medical Laboratory Animal Center, Guangzhou, China) were administered 0.1 mL of a 40% CCl_4_-olive oil solution or only olive oil through oral gavage thrice a week for eight weeks. To evaluate liver fibrosis level of this model, two group of mice (Each group had five control and five CCl_4_ administered mice) were sacrificed after four weeks or eight weeks treatment, respectively. The mice were sacrificed by cervical dislocation 72 h after the final treatment, and the mouse livers were harvested and fixed in 10% buffered formalin for histologic analyses. Other three control mice and three mice with eight weeks CCl_4_ gavage were used for HSC isolation. All animal procedures were approved by the Animal Care and Use Committee of Southern Medical University.

### Histologic analyses

Fixed, paraffin-embedded liver tissues were cut into 4 μm-thick sections and stained with hematoxylin eosin or Sirius Red according to the standard procedures. Fibrosis was staged according to the Ishak scoring system [12].

### Isolation of primary HSCs

Primary mouse HSCs were isolated according to a previously reported protocol [13]. Three control mice and three mice with 8 weeks CCl_4_ gavage induced liver fibrosis were used for HSCs isolation. After the mice were anesthetized with 2% pentobarbital sodium, the abdominal cavity was opened, and the liver was perfused and digested *in situ*. Next, the liver was harvested, minced, homogenized under sterile conditions, and digested further *ex vivo*. The digested liver samples were filtered by passing through a 70 μm steel mesh, and the cells were isolated using density gradient centrifugation.

### RNA sequencing

The RNA samples of primary HSCs (isolated from three control mice and three mice with liver fibrosis) were prepared using TRIzol Reagent (Invitrogen, Waltham, MA) for subsequent RNA sequencing. The quality and concentration of RNA were determined using an Agilent 2100 Bioanalyzer (Agilent, Santa Clara, CA). Next, the purified RNA was polyA-selected and fragmented prior to cDNA synthesis. After the cDNA synthesis and library construction, the libraries were pooled and sequenced on a HiSeq 4000 platform (Illumina, San Diego, CA) in the PE150 mode (Nanjing Vazyme Biotech Company, Ltd). The RNA sequencing data are available from the Gene Expression Omnibus (GEO) under accession numbers GSE149508.

### Real-time quantitative PCR (qPCR)

The total mRNA was isolation from the LX-2 cells untreated/treated with TGF-β1 using TRIzol Reagent (Invitrogen), and 1 μg of mRNA was used for cDNA synthesis with a Transcriptor First Stand cDNA Synthesis Kit (TakaRa, Shiga, Japan) according to the manufacturer’s instructions. Real-time qPCR was performed according to the manufacturer’s instructions using the SYBR Green (Roche, Basel, Switzerland) and LightCycler480 II system (Roche). The samples were run in triplicates, and the results were normalized to the *GAPDH* expression levels using the 2^-ΔΔCT^ method. The gene-specific primers used are listed in the S1 File.

The Student t-test was used to compare the differences between the two groups. The data were expressed as mean ± standard deviation of at least three replicates. The threshold for statistical significance was set at *P* < 0.05. All analyses were performed using Microsoft Excel 2019 (Redmond, WA).

### Public data

The public gene expression data based on the gene chips of HBV- (GSE84044) [14], HCV- (GSE14323) [15], and NAFLD- (GSE49541) [16] associated fibrotic liver tissues were obtained from the National Cancer for Biotechnology Information Gene Expression Omnibus database (GEO).

### Data analysis

For the RNA-seq data analysis, the clean reads that were extracted from the raw reads using HISAT2 were compared to the reference genome to generate the mapped reads [17]. Next, the gene expression was analyzed using Cufflinks [18]. Reads aligned to the reference genome were quantified and normalized to fragments per kilobase of transcript per million fragments mapped (FPKM), and differences between the FPKM values of the activated and quiescent HSCs were compared using Cuffdiff [18]. The public gene expression data were retrieved from the GEO. The Affy [19] and limma [20] packages of R software were used to reanalyze the differentially expressed genes (DEGs). The gene set enrichment analysis (GSEA) was performed using ClusterProfiler [21].

## Results

### Gene expression profiles in HBV-associated fibrotic liver tissues

**Fig 1:**
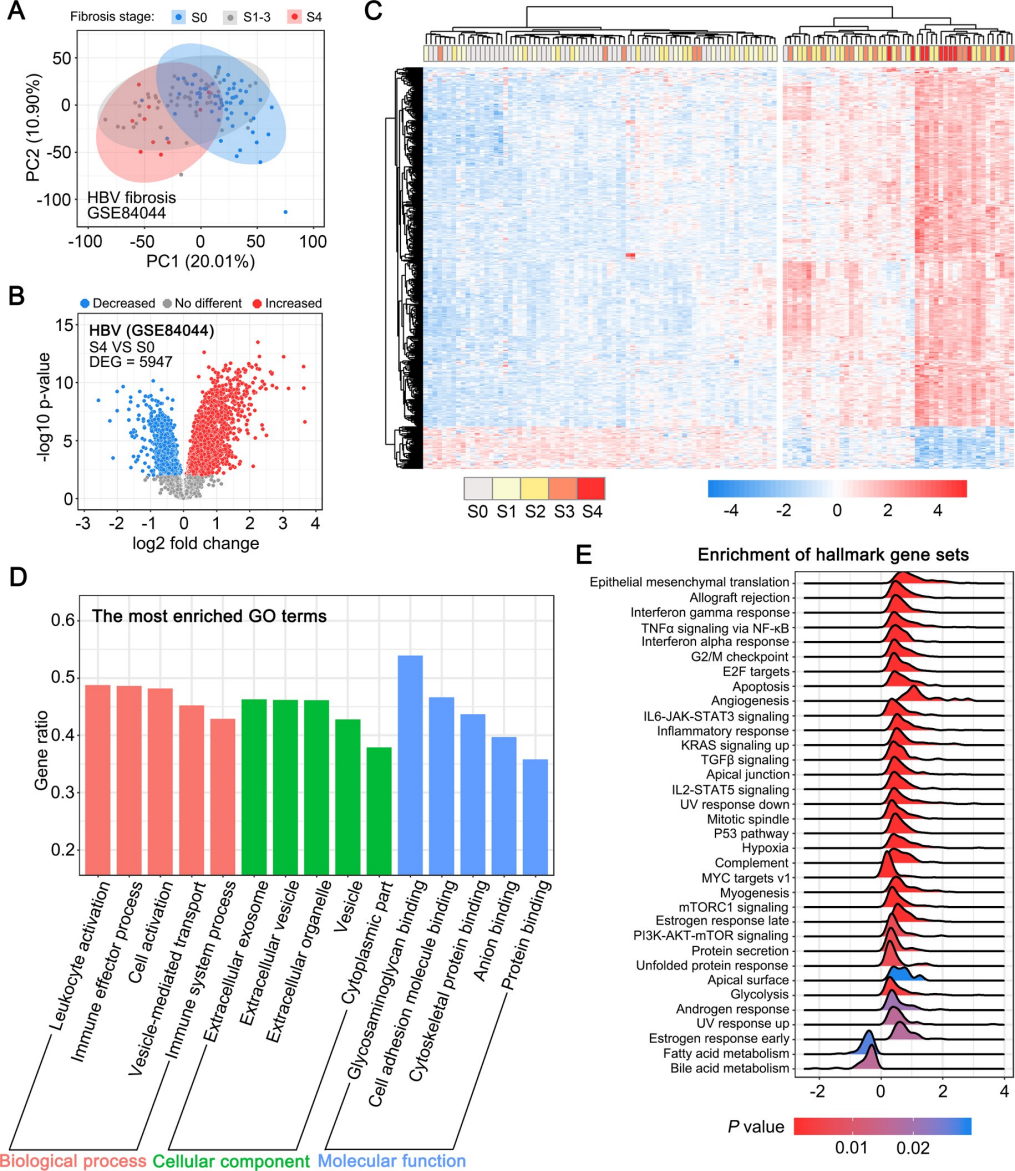
Gene expression profiles in HBV-associated fibrotic liver tissues. (A) Principal component analysis (PCA) of the normalized expression data in liver tissue samples from S0, S1-3, and S4 stage liver fibrosis. (B) Volcano plot depicting the differentially expressed genes (DEGs) between fibrotic liver tissues from stage S4 and S0 (*P* < 0.01). (C) Unsupervised hierarchical clustering of the top 426 DEGs (log_2_ Fold change > 1 or < -1) separated the samples into two major groups. (D) GO analysis of the DEGs between liver fibrotic tissues from stage S4 and S0. Each of the top 5 terms are indicated. (E) A ridgeline plot depicting the significantly enriched signaling pathways revealed by GSEA performed using 50 hallmark gene sets.

As HSC activation is a common process in liver fibrosis of different etiologies, we speculated that HSC activation may contribute to the changes in the expression patterns of genes commonly linked to HBV-, HCV-, and NAFLD-associated liver fibrosis. To this end, we undertook the identification of the common genes by reanalyzing the public gene expression data on HBV-, HCV-, or NAFLD-associated fibrotic liver tissues. First, we reanalyzed the gene expression data of HBV-associated fibrotic liver tissue samples retrieved from the GEO dataset GSE84044, which included data on 124 HBV-infected human liver tissue samples collected at different stages of liver fibrosis (Scheuer's liver fibrosis stage S0-S4). The unsupervised principal component analysis (Fig 1A) revealed the obvious distinction between advanced fibrotic liver tissues (S4 stage) and non-fibrotic liver tissues (S0 stage). Next, we analyzed the DEGs between advanced fibrotic and non-fibrotic liver tissues, and observed that 5,947 genes (*P* value < 0.01) displayed significant differential expression in fibrotic liver tissue samples at S4 versus S0 stage (Fig 1B). The unsupervised hierarchical clustering analysis of the most significant DEGs revealed two distinct groups with minimal overlap (Fig 1C). Samples of fibrotic liver tissues from the S3 and S4 stages tended to cluster together, while the expression profiles of samples from the S1 and S2 stage were considerably similar to those of the non-fibrotic samples (S0 stage). Further, the Gene Ontology (GO) analysis revealed that the DEGs were associated with biological functions or terms, such as immune effector process, extracellular exosome, and cell adhesion molecule binding (Fig 1D). Meanwhile, GSEA performed using MSigDB’s hallmark Gene Sets revealed that a series of gene sets, including those associated with epithelial-mesenchymal translation, IL6-JAK-STAT3 signaling, and the p53 pathway, were enriched in advanced fibrotic liver tissue samples (Fig 1E).

### Gene expression profiles in HCV-associated fibrotic liver tissues

**Fig 2:**
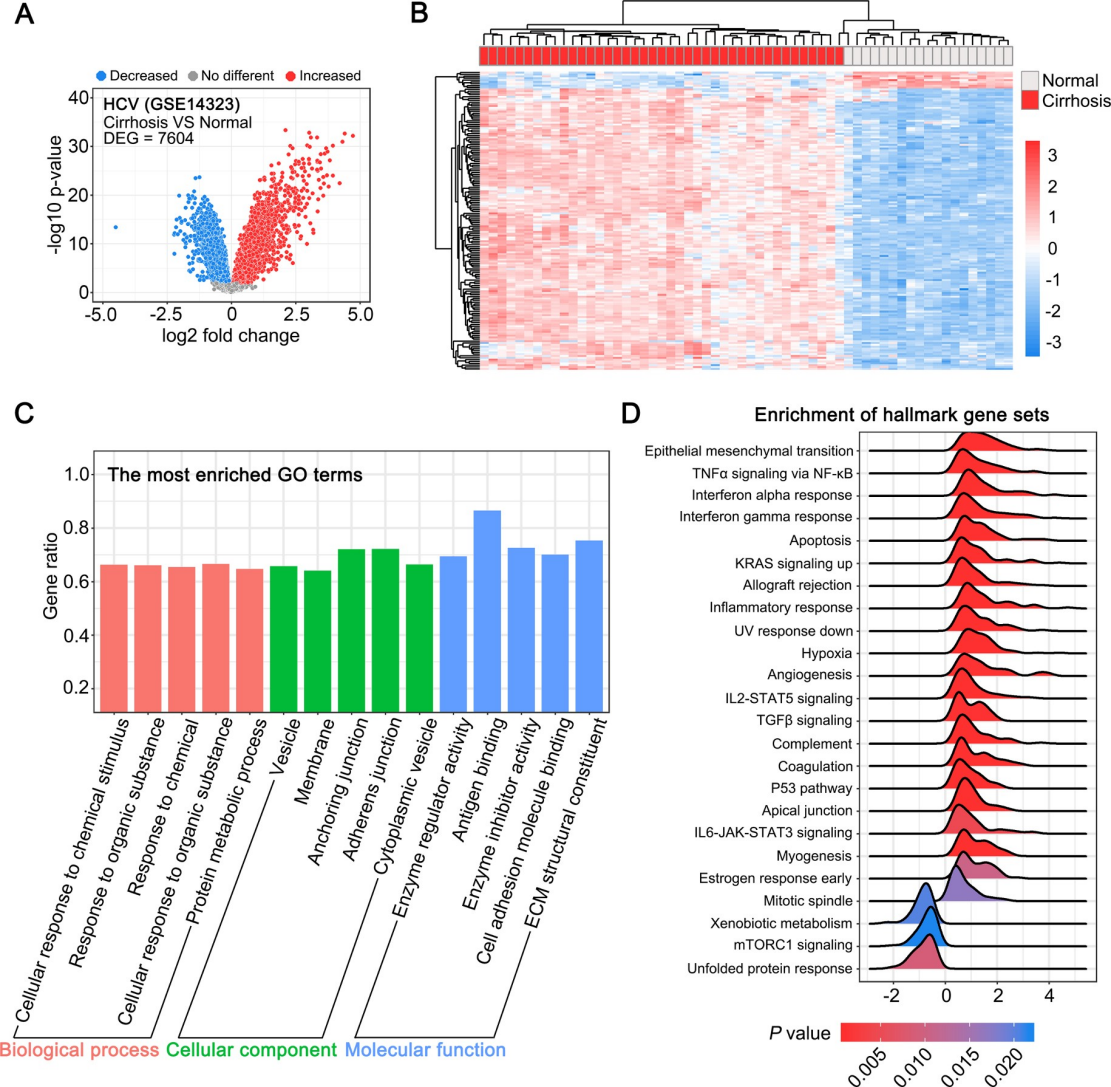
**Gene expression profiles in HCV-associated fibrotic liver tissues.** (A) Volcano plot depicting differentially expressed genes (DEGs) between cirrhotic and normal liver tissues (*P* < 0.01). (B) Unsupervised hierarchical clustering of the top 161 DEGs (log_2_ Fold change > 2 or < -2). (C) GO analysis of the DEGs between the cirrhotic and normal liver tissues. Each of the top 5 terms are indicated. (D) A ridgeline plot depicting the significantly enriched signaling pathways revealed by GSEA performed using 50 hallmark gene sets.

Next, we analyzed the gene expression data for HCV-associated fibrotic liver tissue samples from the GEO dataset GSE14323. We analyzed the data on the DEGs between 41 cirrhotic and 19 normal liver tissue samples from this dataset, and found 7,604 DEGs (Fig 2A). The unsupervised hierarchical clustering analysis of the top 161 DEGs revealed two distinct groups of cirrhotic and normal liver tissue samples (Fig 2B). The GO analysis suggested that the biological functions or terms associated with these DEGs were partially common with the biological functions of the DEGs enriched in HBV-associated fibrotic liver tissues, such as vesicles, and cell adhesion molecule binding (Fig 2C). The major difference is that the immune-associated DEGs were enriched more significantly in HBV-associated fibrotic tissues than in HCV-associated fibrotic tissues. In addition, the GSEA suggested that a series of gene sets, including those associated epithelial-mesenchymal translation, TNFα signaling via NF-κB, IL6-JAK-STAT3 signaling, and p53 pathway, were enriched both in HBV-associated and HCV-associated fibrotic liver tissues (Fig 2D).

### Difference in gene expression between advanced and mild NAFLD-associated fibrotic liver tissues

**Fig 3:**
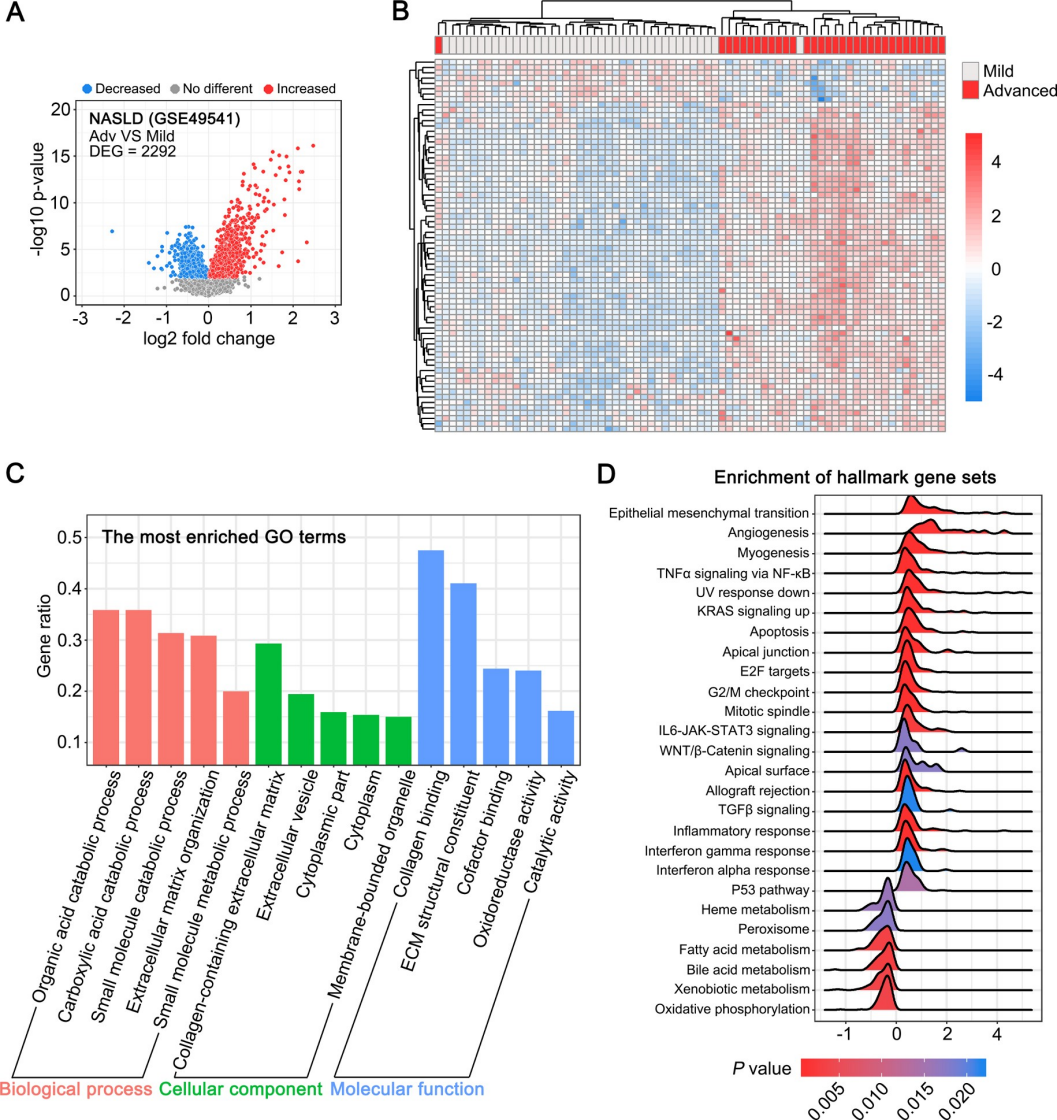
Difference in gene expression profiles between advanced and mild NAFLD-associated fibrotic liver tissues. (A) Volcano plot showing differentially expressed genes (DEGs) between advanced and mild NAFLD-associated fibrotic liver tissues (*P* < 0.01). (B) Unsupervised hierarchical clustering of the top 70 DEGs (log_2_ Fold change > 1 or < -1). (C) GO analysis of the DEGs between advanced and mild NAFLD-associated fibrotic liver tissues. Each of the top 5 terms are indicated. (D) A ridgeline plot depicting the significantly enriched signaling pathways revealed by GSEA performed using 50 hallmark gene sets.

Next, we analyzed the difference in gene expression between 32 advanced (Ishak score 3–4) and 68 mild (Ishak score 0–1) NAFLD-associated fibrotic liver tissues using the data from the GSE49541 dataset. We found 2,292 genes (*P* value < 0.01) exhibiting significant differential expression in mild and advanced NAFLD-associated fibrotic liver tissues (Fig 3A). The unsupervised hierarchical clustering analysis of the top DEGs facilitated the segregation of data related to mild and advanced liver fibrotic tissues (Fig 3B). In contrast to the pattern detected in HBV- and HCV-associated liver fibrosis, the GO analysis indicated that a series of metabolism-related terms, including organic acid catabolic process, carboxylic acid catabolic process, and small molecule metabolic process, were significantly enriched in NAFLD-associated liver fibrosis (Fig 3C). Similarly, the GSEA suggested that certain metabolism-related processes and pathways, such as fatty acid metabolism, bile acid metabolism, heme metabolism, and xenobiotic metabolism, were considerably inhibited in advanced NAFLD-associated fibrotic liver tissues (Fig 3D).

### RNA sequencing of quiescent and activated primary HSCs

**Fig 4:**
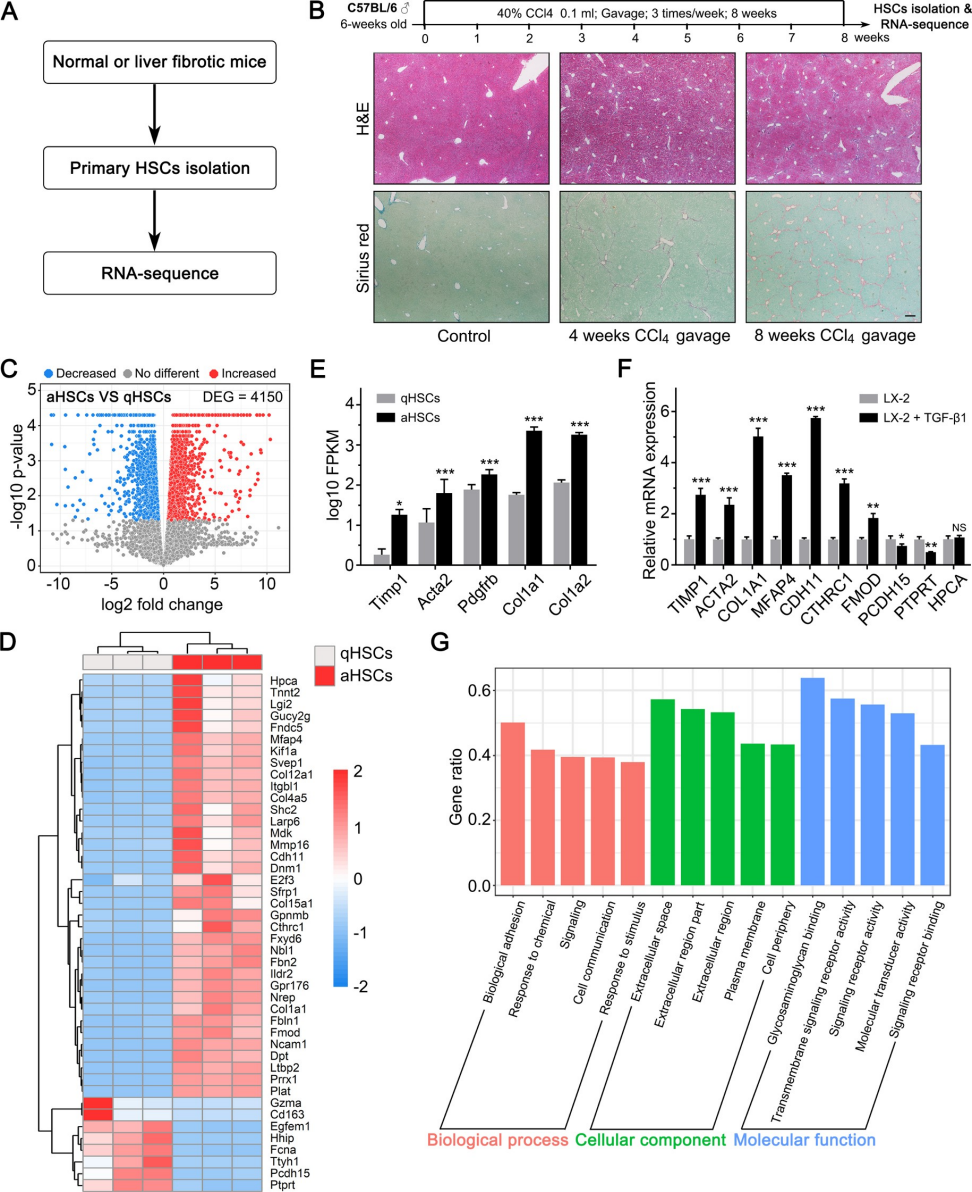
RNA sequencing of quiescent and activated primary HSCs. (A) Schematic of the experimental design. (B) Schematic of the CCl_4_-induced liver fibrosis mouse model establishment. (C) Volcano plot depicting differentially expressed genes (DEGs) between quiescent and activated primary HSCs. (*P* < 0.05). (D) Heatmap and unsupervised hierarchical clustering of the top 45 DEGs. (E) Expression of activated HSC markers in activated HSCs (aHSCs) and quiescent HSCs (qHSCs) detected by RNA-sequencing. (F) Real-time qPCR of the indicated genes in LX-2 cells untreated/treated with 5ng/mL TGF-β1 for 24 hours. (G) GO analysis of the DEGs between quiescent and activated primary HSCs. Each of the top 5 terms are indicated. *, *P* < 0.05; **, *P* < 0.05; ***, *P* < 0.001; NS = not significant.

The above studies were conducted to analyze the gene expression profiles of HBV-, HCV-, and NAFLD-associated fibrotic liver tissues. Next, we performed RNA- sequencing to determine the difference in gene expression patterns in primary quiescent and activated HSCs and analyzed the changes in the expression levels of these genes in the fibrotic liver tissues of different etiologies. Previous studies have suggested that the *in vitro* HSC activation model does not faithfully reproduce the gene expression pattern associated with the activation process observed *in vivo*, whereas the *in vivo* activation of HSC creates represents model that is more comparable to the physiological process [13]. To create an accurate model, we established a CCl_4_-induced liver fibrosis mouse model to isolate *in vivo* activated HSCs (Fig 4A and 4B). The Sirius Red staining revealed that 8 weeks of CCl_4_ gavage (40% CCl_4_ 0.1 mL, thrice per week) induced significant liver fibrosis in the experimental mice (Ishak score 5–6). We isolated the activated HSCs from the liver fibrosis induced mice and quiescent HSCs from control mice. Next, we performed RNA sequencing to identify the DEGs in the activated and quiescent HSCs. The RNA sequencing revealed 4150 DEGs in the quiescent and activated HSCs (Fig 4C and 4D and S2 File). The upregulation of activated HSC markers also confirmed the activation status of the HSCs isolated from the mice with CCl_4_-induced liver fibrosis (Fig 4E). In addition to the well-known activated HSCs markers, some of the most significant DEGs that were identified by RNA sequencing played a critical role in HSC activation or liver fibrosis. Among these genes, *CDH11* [22, 23], *CTHRC1* [24], *FMOD* [25], and *PRRX1* [26] facilitated HSC activation; *Larp6* promoted type I collagen production and was a potential anti-fibrotic target [27, 28]; *HHIP*, a Hedgehog pathway antagonist, inhibited HSC activation and was downregulated in activated HSCs [29]. Moreover, we also evaluated the changes in the expression of the most significant DEGs in TGF-β1-treated LX-2 cells, which is an *in vitro* human HSC activation model (Fig 4F). The data supported the proposition that mouse and human HSCs might undergo similar biological and genetic alterations after activation. Further GO analysis of the DEGs indicated that these were associated with terms such as biological adhesion, extracellular space, and glycosaminoglycan binding (Fig 4G), suggesting that these biological functions or terms might play an important role in HSC activation.

### Overlapping gene expression changes between HSC activation and HBV-, HCV-, and NAFLD-associated liver fibrosis

**Fig 5:**
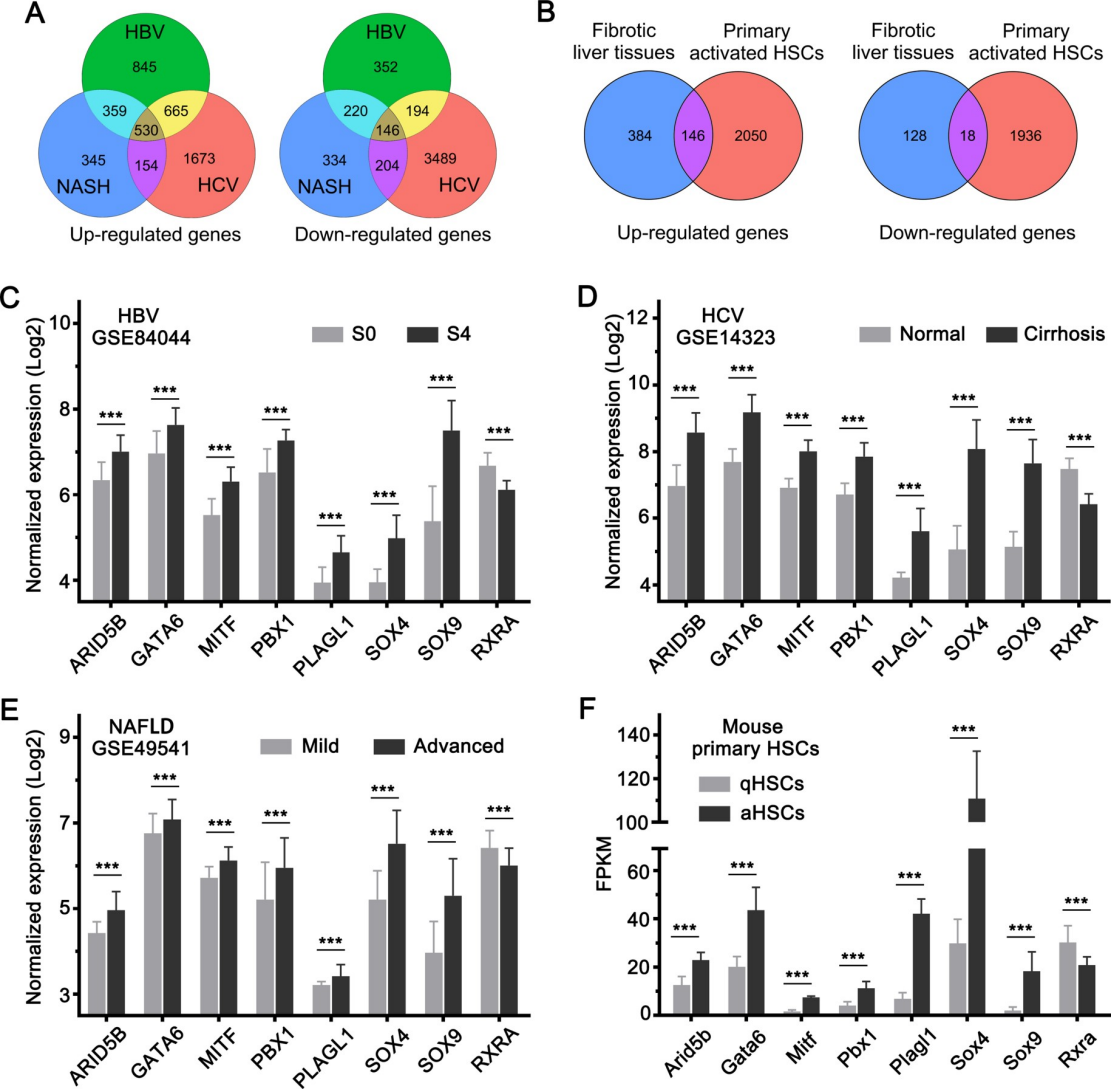
Overlapping gene expression changes in HSC activation and HBV-, HCV-, and NAFLD-associated liver fibrosis. (A) Venn plot depicting the overlapped genes with similar trend of expression changes in HBV-, HCV-, and NAFLD-associated liver fibrosis. (B) Venn plot depicting the overlapped genes with similar trend of expression changes in HSC activation and in HBV-, HCV-, and NAFLD-associated liver fibrosis. (C-F) Expression levels of the transcription factor-encoding genes with similar trend of expression change in (C) HBV-, (D) HCV-, (E) NAFLD-associated liver fibrosis tissues, and in activated HSCs (F). ***, *P* <0.001.

Next, we analyzed the gene expression changes overlapping between HSC activation and liver fibrosis. There were 530 upregulated and 146 downregulated genes overlapping between HBV-, HCV-, and NAFLD-associated fibrotic liver tissues (Fig 5A and S3 File). Among the overlapping genes, 146 upregulated and 18 downregulated genes followed a similar trend of expression change in activated HSCs as well (Fig 5B and S4 File). This finding suggested that these genes were probably the key genes associated with HSC activation that regulate liver fibrosis in different liver diseases. Transcription factors are generally considered to be the key regulators of gene expression and cell function [30]. Therefore, we analyzed the change in the expression of transcription factor-encoding genes from among the overlapped genes. We observed that *ARID5B*, *GATA6*, *MITF*, *PBX1*, *PLAGL1*, *SOX4*, and *SOX9* were upregulated, whereas *RXRA* was downregulated in both activated HSCs and fibrotic liver tissues (Fig 5C–5F). This finding suggested that the transcription factors encoded by these genes probably play a key role in HSC activation and liver fibrosis.

## Discussion

Liver fibrosis is a global health problem without any approved therapies [6]. Therefore, the development of a drug for the treatment of liver fibrosis is necessary and should be undertaken urgently. Activated HSCs play a key role in liver fibrosis, and are commonly used as a target in potential treatment strategies for liver fibrosis. However, the molecular mechanisms underlying the activation of HSCs remain unexplored. The analysis of gene expression changes accompanying HSC activation using high-throughput technology will help elucidate the molecular mechanism underlying HSC activation and liver fibrosis. However, public high-throughput data on differential genetic expression in quiescent and activated HSCs are lacking. In this study, we isolated quiescent and activated HSCs from control mice and mice with liver fibrosis, respectively. We administered CCl_4_ via oral gavage for 8 weeks and induced liver fibrosis in the mouse model to facilitate the isolation of activated HSCs. The CCl_4_-induced liver fibrosis model is widely used in liver fibrosis and cirrhosis research. It has high reproducibility and closely resembles the conditions of human liver fibrosis [31]. In addition to the CCl_4_-induced liver fibrosis model, the common bile duct ligation (BDL) model is also a well-known mouse liver fibrosis model. In contrast to the CCl_4_-induced liver fibrosis model, acute obstructive jaundice and liver injury are induced in the BDL model, and the high mortality rate in the latter is a disadvantage. Moreover, mice exhibit a marked dilatation in the gall bladder upon ligation of the common bile duct, which is an inter-individual variability in gall bladder dilation ensuing a variable parenchymal response [32]. Therefore, we selected the CCl_4_-induced liver fibrosis model for the isolation of *in vivo* activated HSCs.

Liver fibrosis is a complex biological process involving various types of hepatic parenchymal and non-parenchymal cell, and a large number of genes. Different hepatic diseases have different pathologies and affect gene expression variably. However, HSC activation plays a key role and induces similar changes in genes expression in liver fibrosis of different etiologies. Herein, we reanalyzed the public gene expression profile data of HBV-, HCV-, and NAFLD-associated liver fibrosis to detect overlapping gene expression changes, and performed RNA sequencing to determine the changes in gene expression that occur during HSC activation. Next, we integrated the public data and our RNA sequencing data to investigate the specific HSC activation-associated genes that undergo expression modulation synchronously in liver fibrosis resulting from different liver diseases.

Our results suggested that there were 676 overlapping DEGs in HBV-, HCV-, and NAFLD-associated liver fibrosis. Among these, 146 upregulated and 18 downregulated genes underwent synchronous change in expression patterns in the activated HSCs. These genes were suggested to be the key genes associated with HSC activation that facilitate the progression of liver fibrosis in different liver diseases.

In addition, we observed that *ARID5B*, *GATA6*, *MITF*, *PBX1*, *PLAGL1*, *SOX4* and *SOX9*, which are seven transcription factor-encoding genes, were upregulated in activated HSCs as well as in fibrotic liver tissues. Among the corresponding transcription factors, GATA6 [33], SOX4 [34], and SOX9 [35] have been reported to be associated with liver fibrosis. These transcription factors are possibly the key regulators in HSC activation and liver fibrosis.

Our study analyzed the expression of genes associated with HSC activation in liver fibrosis of different etiologies. It would help to elucidating the molecular mechanism underlying HSC activation and liver fibrosis. Although, additional studies are necessary to verify the molecular functions of these genes in liver fibrosis.

## Supporting information

S1 File Primer sequence. The sequence of primers used in the study.
(XLSX)

S2 File Different expression genes between aHSCs and qHSCs. All different expression genes between aHSCs and qHSCs detected by RNA-sequencing.
(XLS)

S3 File Overlapping gene expression changes between HBV-, HCV-, and NAFLD-associated liver fibrosis. (XLSX)

S4 File Overlapping gene expression changes between HSC activation and HBV-, HCV-, and NAFLD-associated liver fibrosis. (XLSX)
